# Association of Hip Fracture With the Use of Walking Assistance Devices Post-surgery: A Retrospective Study Comparing Intertrochanteric and Subtrochanteric Hip Fractures

**DOI:** 10.7759/cureus.36706

**Published:** 2023-03-26

**Authors:** Hasan Nahouli, Daniel Bassil, Aurelie Mailhac, Asdghig Der-Boghossian, Hani Tamim, Muhyeddine Al Taki

**Affiliations:** 1 Department of Orthopedic Surgery, American University of Beirut Medical Center, Beirut, LBN; 2 Clinical Research Institute, American University of Beirut Medical Center, Beirut, LBN

**Keywords:** fragility hip fracture, subtrochanteric hip fracture, intertrochanteric hip fracture, fracture around hip, unstable hip fracture, geriatric hip fracture, elderly trauma, mobility aid

## Abstract

Background

Hip fractures, including intertrochanteric and subtrochanteric fractures, are among the most common types of fractures. The dynamic hip screw (DHS) and the cephalomedullary hip nail (CHN) are the two main techniques used for the fixation of these types of fractures. This study aims to explore the association of the fracture type with the use of walking assistance devices post-surgery, regardless of the fixation technique.

Methodology

This study is a retrospective study based on the review of de-identified patient data from the American College of Surgeons National Surgical Quality Improvement Program database. Patients aged 65 years old or above who underwent fixation procedures for intertrochanteric or subtrochanteric fractures using CHN or DHS techniques were included in this study.

Results

A total of 8,881 patients were included and divided into the following two groups: 876 (9.9%) patients treated for subtrochanteric fracture, and 8,005 (90.1%) patients treated for intertrochanteric fracture. No statistical significance was detected in the use of mobility aid postoperatively between the two groups. When compared to CHN, DHS was noted to be the most employed fixation technique among patients with intertrochanteric fractures. One main finding was that patients who underwent surgery using DHS for intertrochanteric fractures were more likely to use walking assistance devices postoperatively compared to those with subtrochanteric fractures treated with the same fixation technique.

Conclusions

Findings suggest that the use of walking assistance devices post-surgery is independent of the type of fracture and potentially dependent on the fixation technique employed. Future studies focused on the difference in the use of walking assistance devices based on fixation techniques for patients with distinctive sub-types of trochanteric fractures are highly encouraged.

## Introduction

With the increasing life expectancy of the elderly and aging population, hip fractures, including trochanteric fractures, remain among the most common fractures encountered in orthopedic practice [1,2]. Of particular concern are the accompanying implications of such fractures, including morbidity, mortality, and economic burden [3]. Interestingly, hip fractures account for the majority of fracture-related hospitalization, healthcare expenditure, and mortality in women and men above 50 years old [4,5]. From an epidemiological perspective, the total number of hip fractures worldwide is projected to reach 6.3 million fractures in 2050 [6].

Intertrochanteric femur fractures, occurring at the proximal femur distal to the neck extending to the lesser trochanter, account for around 50% of all hip fractures, often caused by low-energy falls such as falling from a standing height [7]. Increased age, female gender, osteoporosis, history of falls, and gait abnormalities have been identified as risk factors for intertrochanteric fractures [8]. On the other hand, subtrochanteric fractures, occurring within 5 cm distal to the lesser trochanter, account for 25% of the proximal femur fractures and often result from ground-level falls in the elderly population [9-11].

The two main techniques used for the fixation of intertrochanteric and subtrochanteric fractures are the dynamic hip screw (DHS) and the cephalomedullary hip nail (CHN) [12]. However, studies show that the differences between the two fixation methods are not statistically significant in terms of mortality, length of hospital stay, length of surgery, blood transfusion requirements, fracture healing, medical complications, re-operations, degree of residual pain, or regain of independence [12].

The timely and adequate treatment of both intertrochanteric and subtrochanteric fractures is crucial in restoring the patient’s function and reducing potentially related morbidity and mortality [1]. This is especially important given that hip fractures have been reported to be associated with a considerable decrease in the overall quality of life, often incurring major disability with regards to pain and loss of functional independence, particularly in the elderly, increasing the burden of disease associated with these types of fractures [13,14].

Despite the wide interest in the quality of life of patients who underwent surgical procedures for intertrochanteric or subtrochanteric fractures, resources remain scarce with regard to the actual use of walking assistance devices post-surgery, as well as factors associated with the latter. Oba et al. suggested that trochanteric fractures are among the factors obstructing the patient’s ability to walk unaided or without a cane at discharge [15].

This study aimed to explore the association of the fracture type (intertrochanteric versus subtrochanteric) with the use of walking assistance devices post-surgery, regardless of the fixation technique used. Understanding whether the fracture type is a predictor for the use of walking assistance devices supports the establishment of focused rehabilitation protocols to prevent potential long-term disability and loss of independence in the elderly, decreasing the burden of these injuries.

## Materials and methods

Study design

This was a retrospective study based on the review of de-identified patient data collected through the American College of Surgeons National Surgical Quality Improvement Program (ACS-NSQIP) database. The ACS-NSQIP database encompasses more than 240 variables on preoperative characteristics and postoperative complications of surgical patients from more than 500 medical centers worldwide. Postoperative variables include 30-day postoperative morbidity and mortality data, along with preoperative risk factors and demographics.

Patient selection

The ACS-NSQIP data from 2016 to 2017 was used to select and include patients aged 65 years old or above, who underwent fixation procedures for intertrochanteric or subtrochanteric fractures as primary surgery, using the following Current Procedural Terminology (CPT) codes: 27245 (hip nail) and 27244 (DHS).

Patients with femoral fractures who underwent procedures other than a fixation with a nail or screw or were younger than 65 years of age were excluded.

Variables

Data collected included demographics and patient information preoperatively as well as postoperative outcomes. Demographics and baseline preoperative variables gathered included gender, age, race, body mass index (BMI), tobacco use, functional status and American Society of Anesthesiologists (ASA) class, and comorbidities (e.g., diabetes and hypertension, chronic heart failure, severe chronic obstructive pulmonary disease). In addition, information about mean operative time, fixation method used, anemia (based on gender-specific hematocrit levels), and preoperative laboratory values were gathered. Other preoperative variables gathered were the use of walking assistance devices by the patient; the presence of delirium, dementia, and pressure sores; and whether the patient was taking any type of bone-protective medication. The fracture type with which the patient presented for surgery was recorded. The patients were accordingly divided into the following two groups: group 1 representing patients with subtrochanteric fractures, and group 2 representing patients with intertrochanteric fractures.

Regarding the postoperative outcomes, the patients were evaluated for 30-day postoperative mortality and composite morbidity including all specific morbidities. The specific morbidities included postoperative cardiac, respiratory (severe respiratory distress or need for ventilation), urinary (renal problem or urinary tract infection), central nervous system (CNS) morbidities, as well as the development of thromboembolism, wound complications, and sepsis.

Other procedure-specific postoperative outcome data were gathered. These included the postoperative use of any type of walking assistance device as the primary outcome; the ability to weight-bearing on postoperative day one; and delirium, pressure sores, or pathologic fracture. Postoperative data were collected including postoperative deep vein thrombosis (DVT) prophylaxis, bone-protecting medication prescription, and whether the patient was offered a standardized hip care program as rehabilitation. Data on postoperative discharge residency were collected and categorized into either care facility (i.e., any type of facility whether skilled or not, including hospital, or acute care center) or home.

Statistical analysis

The statistical analyses were performed using the Statistical Analysis Software (SAS; SAS Institute Inc., Cary, NC, USA). Descriptive analyses were presented as frequencies (n) and percentages (%) for categorical variables and as means and standard deviations (SDs) for continuous variables. These were compared using the chi-square/Fisher test and independent t-test, respectively.

Multivariate logistic regression analyses were done to control for potentially confounding effects of patient characteristics and calculate the adjusted odds ratios (ORs). The ORs were calculated for walking assistance device use and other postoperative outcomes at a 95% confidence interval (CI) and compared between the two fracture groups. Furthermore, the ORs of the walking assistance device use were stratified by age, BMI, and mean operative time. Statistical significance was determined for p-values <0.05.

## Results

A total of 8,881 patients were included in this study. These were divided into two groups, namely, group 1 which included 876 (9.9%) patients who underwent a fixation procedure for subtrochanteric fracture as primary surgery, and group 2 which included 8,005 (90.1%) patients who underwent a fixation procedure for intertrochanteric fracture as primary surgery.

Baseline characteristics

Preoperative baseline characteristics of included patients are presented in Table 1. The majority of the sample were females (72.9%) and of the white race (94.7%). The mean age for group 2 (81.56 ± 7.76) was noted to be significantly higher than that of group 1 (82.78 ± 7.29), with a p-value <0.01. Similarly, the mean weight in kg was significantly higher for group 1 (154.20 ± 40.28) compared to group 2 (143.8 ± 37.66) with a p-value <0.01. Patients with subtrochanteric fractures had significantly higher BMI than those with intertrochanteric fractures. The majority of included patients were functionally independent presurgery (77.9%), with a significantly higher percentage of functionally independent patients in group 1 compared to group 2 (p = 0.01). The mean total operative time was significantly higher for patients with subtrochanteric fractures compared to those with intertrochanteric fractures. Perioperative transfusion (p = 0.03) and disseminated cancer (p < 0.01) were significantly more prevalent among patients with subtrochanteric fractures compared to those with intertrochanteric fractures.

**Table 1:** Preoperative baseline characteristics reported as frequency and percentage for categorical data and mean ± SD for continuous data with p-value <0.05 showing a statistical significance between group 1 and group 2. Mean ± SD is denoted by #. Group 1: subtrochanteric fractures. Group 2: intertrochanteric fractures.

Preoperative characteristics	All (n = 8,881)	Group 1 (n = 876)	Group 2 (n = 8,005)	P-value
Age (years)^#^	82.78 ± 7.29	81.56 ± 7.76	82.91 ± 7.22	<0.01
Gender				
Male	2,410 (27.14%)	236 (26.94%)	2,174 (27.16%)	0.90
Female	6,471 (72.86%)	640 (73.06%)	5,831 (72.84%)	
Race				
White	6,176 (94.72%)	520 (90.43%)	5,656 (95.14%)	<0.01
Black	171 (2.62%)	27 (4.70%)	144 (2.42%)	
Other	173 (2.65%)	28 (4.87%)	145 (2.44%)	
Weight (kg)^#^	144.84 ± 38.04	154.20 ± 40.28	143.80 ± 37.66	<0.01
Body mass index^#^	24.96 (5.68%)	26.45 (6.15%)	24.8 (5.61%)	<0.01
<18.5 kg/m^2^	649 (8.58%)	32 (4.43%)	617 (9.02%)	<0.01
18.5–24.9 kg/m^2^	3,591 (47.46%)	298 (41.22%)	3,298 (48.12%)	
25–29.9 kg/m^2^	2,122 (28.05%)	234 (32.37%)	1,888 (27.59%)	
≥30 kg/m^2^	1204 (15.91%)	159 (21.99%)	1,045 (15.27%)	
Tobacco use in the past year	782 (8.81%)	74 (8.45%)	708 (8.84%)	0.75
Diabetes on medication	1,615 (18.18%)	177 (20.21%)	1,438 (17.96%)	0.11
Hypertension on medication	6,143 (69.17%)	587 (67.01%)	5,556 (69.41%)	0.15
Steroid use for a chronic condition	463 (5.21%)	49 (5.59%)	414 (5.17%)	0.58
Systemic sepsis in the previous 48 hours	794 (8.94%)	84 (9.59%)	710 (8.87%)	0.49
Functional status				
Independent	6866 (77.89%)	700 (80.74%)	6,166 (77.58%)	0.01
Partially dependent	1,700 (19.29%)	137 (15.8%)	1,563 (19.67%)	
Dependent	249 (2.82%)	30 (3.46%)	219 (2.76%)	
American Society of Anesthesiologists class				
I–II	1,325 (14.95%)	150 (17.14%)	1,175 (14.71%)	0.06
III–V	7,536 (85.05%)	725 (82.86%)	6,811 (85.29%)	
Type of Anesthesia				
General	6,028 (67.88%)	576 (65.75%)	5,452 (68.11%)	0.35
Spinal/Epidural	2,084 (23.47%)	217 (24.77%)	1,867 (23.32%)	
Other	769 (8.66%)	83 (9.47%)	686 (8.57%)	
Days from admission to Operation (days)^#^	1.22 (4.92%)	1.13 (1.36%)	1.23 (5.16%)	0.16
Mean total operation time	56.13 (38.72%)	84.49 (56.40%)	53.03 (34.90%)	<0.01
<60 minutes	5,897 (66.4%)	309 (35.27%)	5,588 (69.81%)	<0.01
60–120 minutes	2607 (29.35%)	416 (47.49%)	2191 (27.37%)	
>120 minutes	377 (4.25%)	151 (17.24%)	226 (2.82%)	
Perioperative transfusion	520 (5.86%)	66 (7.53%)	454 (5.67%)	0.03
Disseminated cancer	178 (2.00%)	52 (5.94%)	126 (1.57%)	<0.01
Preoperative white blood cell count				
≤11,000 cells/µL	5,919 (66.94%)	599 (68.85%)	5,320 (66.73%)	0.21
>11000 cells/µL	2,923 (33.06%)	271 (31.15%)	2,652 (33.27%)	
Hematocrit				
No anemia	2,836 (32.02%)	295 (33.79%)	2,541 (31.83%)	0.34
Mild anemia	2,130 (24.05%)	195 (22.34%)	1,935 (24.24%)	
Moderate-to-severe anemia	3,890 (43.93%)	383 (43.87%)	3,507 (43.93%)	
Preoperative dementia				
No	6,230 (70.15%)	673 (76.83%)	5,557 (69.42%)	<0.01
Yes	2,651 (29.85%)	203 (23.17%)	2,448 (30.58%)	
Preoperative delirium				
No	7,816 (88.01%)	802 (91.55%)	7,014 (87.62%)	<0.01
Yes	1,065 (11.99%)	74 (8.45%)	991 (12.38%)	
Pre-fracture bone protection medication prescription				
No	5,946 (66.95%)	539 (61.53%)	5,407 (67.55%)	<0.01
Yes	2,935 (33.05%)	337 (38.47%)	2,598 (32.45%)	
Preoperative use of walking assistance devices				
No	3,753 (43.63%)	386 (45.57%)	3,367 (43.42%)	0.24
Yes	4,848 (56.37%)	461 (54.43%)	4,387 (56.58%)	
Preoperative pressure sore				
No	8,615 (97.00%)	851 (97.15%)	7,764 (96.99%)	0.92
Yes	266 (3.00%)	25 (2.85%)	241 (3.01%)	
Fixation technique				
Hip nail	7,190 (80.96%)	761 (86.87%)	6,429 (80.31%)	<0.01
Dynamic hip screw	1,691 (19.04%)	115 (13.13%)	1,576 (19.69%)	

Regarding preoperative morbidities, both dementia and delirium were significantly more prominent among patients with intertrochanteric fractures compared to patients with subtrochanteric fractures. In terms of medication use, significantly more patients with subtrochanteric fractures were taking pre-fracture bone-protection medication (p < 0.01). In terms of surgical procedure, hip nailing was significantly more frequently performed for patients with subtrochanteric fractures while the application of a DHS was performed more for patients with intertrochanteric fractures.

Postoperative outcomes

Results of multivariate logistic regression shown in Table 2 highlight that patients with intertrochanteric fractures are more likely to bear weight as tolerated one day after surgery (OR = 1.69, 95% CI = 1.45-1.98; p < 0.01), and to receive medical DVT prophylaxis (OR = 1.23, 95% CI = 1.06-1.44; p < 0.01) compared to patients with subtrochanteric fractures. Patients with intertrochanteric fractures were also noted to be less likely to have pathological fractures compared to those with subtrochanteric fractures (OR = 0.11, 95% CI = 0.08-0.16; p < 0.01). No statistical significance was detected in the use of walking assistance devices postoperatively between those who underwent surgery for subtrochanteric fracture and those who underwent surgery for intertrochanteric fracture.

**Table 2:** Multivariable logistic regression outcomes of postoperative use of walking assistance devices and other hip fracture surgery outcomes comparing group 1 and group 2 (p < 0.05 is statistically significant). n (%): frequency (percentage); OR: adjusted odds ratio; CI: confidence interval; DVT: deep vein thrombosis continued 28 days postoperatively; $ “Home” refers to being discharged to home or any facility that can be considered as home; while “Care facility” refers to a skilled and unskilled care facility, hospital, or any type of specialized care other than home.

Postoperative outcomes	Group 1, n (%)	Group 2, n (%)	P-value	Group 1 versus Group 2	
				OR (95% CI)	P-value
Use of walking assistance devices	731 (83.45%)	6,519 (81.44%)	0.15	0.90 (0.74-1.10)	0.32
Weight-bearing as tolerated on postoperative day 1	501 (63.26%)	5,414 (73.71%)	<0.01	1.69 (1.45-1.98)	<0.01
Medical DVT prophylaxis	532 (60.73%)	4,902 (61.24%)	0.77	1.23 (1.06-1.44)	<0.01
Standardized hip fracture care program	499 (56.96%)	4,262 (53.24%)	0.04	0.98 (0.84-1.14)	0.77
Pathological fracture	105 (11.99%)	111 (1.39%)	<0.01	0.11 (0.08-0.16)	<0.01
Delirium	210 (23.97%)	2,356 (29.43%)	<0.01	1.10 (0.92-1.32)	0.31
Bone-protection medication prescription	486 (55.48%)	3,964 (49.52%)	<0.01	0.92 (0.77-1.10)	0.36
Pressure sore	48 (5.48%)	429 (5.36%)	0.87	0.95 (0.70-1.30)	0.76
Discharge residency^$^					
Care facility	349 (48.14%)	2,976 (46.52%)	0.41	1.06 (0.89-1.25)	0.52
Home	376 (51.86%)	3,421 (53.48%)			

Results of multiple logistic regression analysis shown in Table 3 indicate that transfusion is less likely to occur among patients with intertrochanteric fractures compared to those with subtrochanteric fractures (OR = 0.64, 95% CI = 0.54-0.75; p < 0.01). No statistically significant difference in the occurrence of other postoperative outcomes was noted between the two groups for all other postoperative morbidities or mortality.

**Table 3:** Comparison of the rates and odds ratios of postoperative mortality and morbidities between group 1 and group 2 (p-value <0.05 shows statistical significance). n (%): frequency (percentage); OR: adjusted odds ratio; CI: confidence interval

Postoperative outcomes	Group 1	Group 2		OR (95% CI)	
	n (%)	n (%)	P-value	Group 1 vs. Group 2	P-value
Mortality	29 (3.31%)	381 (4.76%)	0.05	1.34 (0.90-2.00)	0.15
Return to OR (related)	18 (2.05%)	145 (1.81%)	0.59	0.90 (0.55-1.47)	0.67
Composite morbidity	66 (7.53%)	621 (7.76%)	0.89	1.08 (0.82-1.43)	0.58
Cardiac	24 (2.74%)	241 (3.01%)	0.75	1.16 (0.75-1.81)	0.51
Respiratory	7 (0.80%)	78 (0.97%)	0.72	1.24 (0.56-2.75)	0.60
Urinary	8 (0.91%)	55 (0.69%)	0.40	0.71 (0.33-1.50)	0.37
Central nervous system	6 (0.68%)	75 (0.94%)	0.58	1.37 (0.60-3.16)	0.46
Wound	3 (0.34%)	26 (0.32%)	0.76	1.35 (0.38-4.74)	0.64
Thromboembolism	18 (2.05%)	150 (1.87%)	0.69	0.88 (0.54-1.44)	0.61
Sepsis	13 (1.48%)	120 (1.50%)	1.00	0.98 (0.55-1.75)	0.95
Transfusion	412(47.03%)	2691 (33.62%)	<0.01	0.64 (0.54-0.75)	<0.01

When data on postoperative walking assistance device use was stratified by baseline characteristics and studied between the two groups (Table 4), no statistical difference was detected with regard to using postoperative walking assistance devices in those who underwent hip nailing. On the other hand, patients who underwent surgery using DHS in the intertrochanteric fracture group were more likely to use walking assistance devices postoperatively compared to those in the subtrochanteric fracture group treated with the same fixation technique. All other strata showed no difference in the postoperative use of walking assistance devices.

**Table 4:** Stratification of the postoperative use of walking assistance devices by baseline characteristics (p < 0.05 is considered statistically significant). n: frequency; OR: adjusted odds ratio; CI: confidence interval

Postoperative use of walking assistance devices	Group 1	Group 2		Group 1 versus Group 2	
	n (%)	n (%)	P-value	OR (95% CI)	P-value
Gender					
Male	356 (90.36%)	1,818 (90.18%)	1.00	1.11 (0.76-1.63)	0.59
Female	1,130 (91.35%)	4,701 (89.82%)	0.11	0.84 (0.67-1.06)	0.14
Body mass index					
<18.5 kg/m^2^	5 (3.36%)	27 (5.40%)	0.39	0.57 (0.21-1.56)	0.27
18.5–24.9 kg/m^2^	58 (8.88%)	240 (8.17%)	0.53	1.03 (0.75-1.40)	0.87
25–29.9 kg/m^2^	32 (9.14%)	202 (11.40%)	0.26	0.90 (0.65-1.24)	0.52
≥30 kg/m^2^	24 (10.43%)	135 (13.86%)	0.19	0.76 (0.48-1.23)	0.26
Tobacco use in the past year	9 (0.74%)	65 (9.57%)	1.00	1.02 (0.46-2.25)	0.96
Mean total operation time (minute)					
<60 minutes	51 (4.67%)	258 (5.37%)	0.37	0.88 (0.63-1.22)	0.43
120–60 minutes	69 (14.5%)	347 (16.28%)	0.37	0.93 (0.69-1.25)	0.62
>120 minutes	25 (39.68%)	126 (40.13%)	1.00	1.00 (0.56-1.77)	0.99
Fixation technique					
Hip nail	137 (9.79%)	624 (10.78%)	0.31	0.96 (0.78-1.18)	0.67
Dynamic hip screw	8 (3.45%)	107 (7.33%)	0.03	0.44 (0.21-0.92)	0.03

## Discussion

This study aimed to explore the association of the fracture type (intertrochanteric versus subtrochanteric) with the use of walking assistance devices post-surgery.

When compared to CHN, our results showed that DHS was the most employed fixation technique among patients with intertrochanteric fractures. This finding is consistent with Sambandam et al. who reported that nailing cannot be routinely recommended for stable intertrochanteric fractures, for which DHS should be considered the standard of care [16].

One main finding of our study was that patients who underwent surgery using DHS for intertrochanteric fractures were more likely to use walking assistance devices postoperatively compared to those with subtrochanteric fractures treated with the same fixation technique. While there is an evident dearth of studies in the literature exploring the use of walking assistance devices based on different types of fractures, existing studies are more likely to focus on the variation in the use of walking assistance devices based on different fixation techniques within specific types of fractures [17,18]. Having said that, Pajarinen et al. stated that the use of proximal femoral nails in patients with pertrochanteric femoral fractures provided faster restoration of walking ability compared to the use of DHS [17]. Similarly, Pradhan et al. stated that the proximal femoral nail (PFN) is a weight-sharing implant compared to DHS which is a weight-bearing implant, which could also explain our findings [18].

Trochanteric fractures are common in elderly populations due to several reasons, including poor bone quality and higher chances of falls [19,20], yet, it is worth noting that the mean age of our sample was reported as 82.78 ± 7.29, which is relatively higher than the mean age of many other samples with trochanteric fractures reported in previous studies [19,21,22]. For example, the mean age in studies by Sahoo et al. was 73 years for a sample of 30 elderly with trochanteric fractures [19], by Puram et al. was 72 years for a sample of 102 patients with intertrochanteric fractures [21], and by Sinno et al. was 78.6 years for a sample of 48 patients with intertrochanteric fractures [22].

While this study contributes to the current knowledge in orthopedic practice, several limitations exist. One of the primary limitations is that the ACS-NSQIP database only tracks morbidity and mortality within 30 days after surgery; therefore, any complication that occurred after this timeframe was not considered as part of this analysis, and, consequently, long-term follow-up was not possible. Additionally, although comorbidities were included in our analysis, the ACS-NSQIP treats the comorbidity as a binary variable with no further indications regarding the severity of the comorbidity. Finally, the database does not include data from non-contributing medical institutions, which may have led to a disproportionate contribution to the ACS-NSQIP database given the high costs associated with such a contribution. Despite these limitations, basing our study on data from the ACS-NSQIP has ensured that the data analyzed are precise and accurate given the database’s validity and reliability.

## Conclusions

This study aimed to explore the association of the fracture type (intertrochanteric versus subtrochanteric) with the use of walking assistance devices post-surgery, regardless of the fixation technique used. However, findings suggest that the use of walking assistance devices post-surgery is independent of the type of fracture and potentially dependent on the fixation technique employed. Future studies focused on the difference in the use of walking assistance devices based on fixation techniques for patients with distinctive sub-types of trochanteric fractures are highly encouraged.
